# The mental health and well-being effects of wildfire smoke: a scoping review

**DOI:** 10.1186/s12889-022-14662-z

**Published:** 2022-12-05

**Authors:** David P. Eisenman, Lindsay P. Galway

**Affiliations:** 1 David Geffen School of Medicine at UCLA, UCLA Fielding School of Public Health, Center for Healthy Climate Solutions and Center for Public Health and Disasters, 1100 Glendon Avenue, Suite 850-878, Los Angeles, CA 90024 USA; 2 Lakehead University Department of Health Sciences, 955 Oliver Road, Thunder Bay, ON P7B 5E1 Canada

**Keywords:** Wildfire smoke, Landscape fire, Wildfire, Bushfire, Haze, Mental health, Well-being, Scoping review

## Abstract

**Background:**

Smoke from wildfires is a growing public health risk due to the enormous amount of smoke-related pollution that is produced and can travel thousands of kilometers from its source. While many studies have documented the physical health harms of wildfire smoke, less is known about the effects on mental health and well-being. Understanding the effects of wildfire smoke on mental health and well-being is crucial as the world enters a time in which wildfire smoke events become more frequent and severe. We conducted a scoping review of the existing information on wildfire smoke’s impact on mental health and well-being and developed a model for understanding the pathways in which wildfire smoke may contribute to mental health distress.

**Methods:**

We conducted searches using PubMed, Medline, Embase, Google, Scopus, and ProQuest for 1990–2022. These searches yielded 200 articles. Sixteen publications met inclusion criteria following screening and eligibility assessment. Three more publications from the bibliographies of these articles were included for a total of 19 publications.

**Results:**

Our review suggests that exposure to wildfire smoke may have mental health impacts, particularly in episodes of chronic and persistent smoke events, but the evidence is inconsistent and limited. Qualitative studies disclose a wider range of impacts across multiple mental health and well-being domains. The potential pathways connecting wildfire smoke with mental health and well-being operate at multiple interacting levels including individual, social and community networks, living and working conditions, and ecological levels.

**Conclusions:**

Priorities for future research include: 1) applying more rigorous methods; 2) differentiating between mental illness and emotional well-being; 3) studying chronic, persistent or repeated smoke events; 4) identifying the contextual factors that set the stage for mental health and well-being effects, and 5) identifying the causal processes that link wildfire smoke to mental health and well-being effects. The pathways model can serve as a basis for further research and knowledge synthesis on this topic. Also, it helps public health, community mental health, and emergency management practitioners mitigate the mental health and well-being harms of wildfire smoke.

## Background

Climate change is leading to increased frequency, duration, and severity of wildfires, with longer fire seasons too [1, 2, 3, 4, 5]. Wildfires are a growing public health risk due to the enormous amount of smoke related pollution produced, including carbon monoxide, nitrous oxide, volatile organic compounds and particulate matter less than 2.5 μm in diameter (PM_2.5_), which are mixtures of solid and liquid particles that are suspended in the air. Wildfire smoke can travel thousands of kilometers from the source polluting the air over distant populations for weeks and months [6]. For example, long-range transport of pollutants from Canadian forest fires contributed to peak PM_2.5_ and PM_10_ values of 200 and 650 μg/m^3^ in Baltimore, Maryland [7]. These levels were 17 times higher than the U.S. Environmental Protection Agency’s National Ambient Air Quality standard for PM_2.5_ and four times higher than the PM_10_ standard. Wildfire smoke episodes are expected to increase as climate change brings warming temperatures and drier weather, leading to more smoke events even several thousand miles away from the source fires. Climate change models predict a 55% increase in wildfire-related pollution from PM_2.5_ (under Representative Concentration Pathway [RCP] 4.5) to a 190% increase (under RCP 8.5) [8].

Many studies have examined and documented the physical health harms of wildfire smoke exposure including cardiovascular, respiratory, and neuro-cognitive effects [9]. Studies are also documenting these effects occur far from the wildfire. Cardiorespiratory hospitalization rates increased across the mid-Atlantic and Northeastern United States from fires thousands of kilometers away in Quebec Province, Canada [10]. O’Dell and colleagues reported that while most large fires occur in the western U.S., a majority of the attributable mortality and asthma morbidity occurs in the eastern U.S., due to the higher population density there [11]. Over 1.4 million wildfires have occurred in the U.S. since 2000, causing more than 15,000 fatalities per year due to harmful emissions [8]. These harms are unevenly distributed across subpopulations, with children, the elderly, women, racial and ethnic minorities, those living in low socioeconomic areas, and those with preexisting comorbidities at greatest risk [12, 13, 14, 15, 16, 17, 18]. Research has not adequately elucidated the mechanisms for these differences in effects [18]. While the increased risk faced by children and the elderly may be imparted by physical and physiological susceptibility, disparities experienced across lines of social advantage and disadvantage may result from constructed social and environmental reasons [19]. In one intriguing study, Shrestha and colleagues investigated indoor and outdoor levels of PM2.5 in low-income homes at the wildland-urban interface in Colorado on days when ambient air pollution was elevated from short- and long-range wildfire smoke [20]. Controlling for indoor impacts from cooking, indoor/outdoor ratios of PM_2.5_ were slightly less than one, indicating only slightly higher levels outdoors than indoors due to the use of open windows in these homes for ventilation. The authors hypothesized that poorer quality and older construction and lack of air conditioning, all related to lower socioeconomic status, were associated with these higher rates of indoor air pollution. Rural and Indigenous communities are also at heightened risk for health and well-being impacts of exposure to wildfire smoke [21, 22] possibly due to closer proximity to wildfires [23], longer residential tenure in smoke-exposed geographies, reduced access to services, and higher levels of comorbidities [24]. In Canada, approximately 60% of First Nation reserves are located within regions that are at high risk from wildfire events [25].

The increasing intensity of wildfire events and duration of wildfire seasons, combined with current and anticipated wildfire-related health impacts underscores the importance of understanding the full range of potential health effects including impacts on mental health and well-being [26]. Herein, we define mental health following the American Psychological Association as “a state of mind characterized by emotional well-being, good behavioral adjustment, relative freedom from anxiety and disabling symptoms, and a capacity to establish constructive relationships and cope with the ordinary demands and stresses of life” [27]. The mental health effects of climate change generally, and wildfire smoke specifically, have not been adequately examined compared to physical health endpoints. Studies documenting the risk of adverse mental health consequences after experiencing a wildfire have focused on the traumatic experience of being in a life-threatening experience, losing property, the stress of evacuation, and the stress of recovery [28]. Less attention has focused on the effects of smoke on mental health and well-being. Exploring this relationship is even more pertinent considering the growing body of research documenting the negative effects of air pollution on mental health, including depression, anxiety, suicide, and psychological distress [29, 30, 31, 32, 33, 34, 35]. Understanding these effects of wildfire smoke is crucial as the world enters a time in which wildfire smoke events can spread hundreds of miles past the immediate burn for prolonged periods of time and the interconnection between the physical and mental well-being effects of smoke is increasingly recognized [36].

We conducted a scoping review on the impacts of wildfire smoke on mental health and well-being. Our aim was to review the existing information on wildfire smoke’s impact on mental health and well-being and to develop a model for understanding the ways in which wildfire smoke may contribute to mental health distress. For the purposes of this review, wildfire smoke (referred to as bushfire smoke in some contexts) was defined broadly as smoke from the landscape including both wild lands and agricultural lands. This broad definition encompasses various sources of smoke including from forests and peatland fires. It permitted a more wide-ranging and comprehensive review of the literature and was made necessary by existing studies’ broad definitions.

## Methods

We followed the five phase process outlined by Arksey and O’Malley for conducting scoping reviews [37] which includes: (1) identifying the research objectives/questions, (2) identifying relevant publications, (3) selection of publications, (4) data charting, and (5) collating, summarizing, and reporting the results.

Librarians at the U.S. National Academies of Science, Engineering, and Medicine and Lakehead University conducted systematic electronic database searches using Google, Scopus, PubMed, Medline, Embase and ProQuest Research Library from January 1990 to February 2022 to identify scientific papers, journal articles, book chapters, and reports related to mental health and well-being impacts of wildfire smoke exposure [38]. This date range was selected to “balance feasibility with breadth and comprehensiveness” as outlined by Levac [38] and based on our general knowledge of the scholarly research in this realm (it is noteworthy that only one article identified in our database searches was published between 1990 and 2004, justifying this decision [39]). The search was conducted using a combination of multiple keywords including “environmental exposure”, “smoke”, “mental health”, “depression”, “anxiety”, “psychological symptoms”, and “psychological distress”. The search was limited to studies in the English language. After duplicates were removed, titles, abstracts and full texts were screened for relevance. Exclusion criteria were applied (see Table 1). Importantly, publications that did not explicitly examine the relationship between mental health and/or well-being and wildfire smoke events/exposures were excluded (i.e., exclusive focus on wildfire evacuations without smoke exposure). Questionable cases were resolved through discussion among the authors. In all cases, we searched the citations of final eligible articles to ensure the inclusion of all the relevant articles. Lastly, we contacted experts to locate unpublished reports from the gray literature. All evaluation of results from these studies was based on the authors’ interpretation of the reported findings in each paper.

**Table 1:** Inclusion and exclusion criteria used in review

*Inclusion criteria*	*Exclusion criteria*
Empirical peer-reviewed research	Conference abstracts or proceedings, protocols/frameworks, commentaries, articles in media, editorials, literature reviews without a search strategy, letters to the editor, book reviews, textbooks, replies from author, erratum, thesis, or opinion pieces
Publication explicitly examined the relationship between mental health and/or well-being and wildfire smoke events and/or exposures	Publication does not explicitly examine the relationship between mental health and/or well-being and wildfire smoke events/exposures
Publications discussed mental health and/or well-being related impacts or outcomes in humans	Publication is an animal study
English language	Publications in languages other than English

**Fig. 1:**
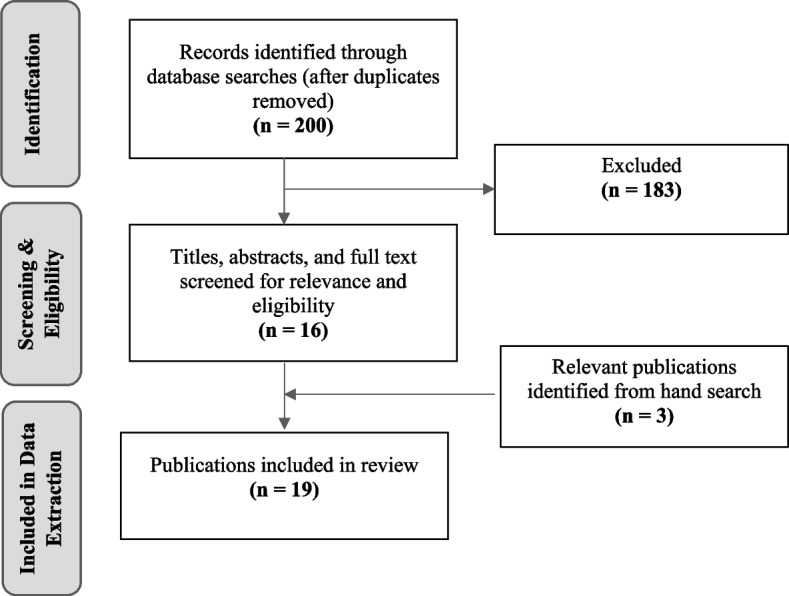
Flow diagram of records identified through database searches, relevance and eligibility screening, and hand searches

The database searches yielded a total of 200 articles (see Fig. 1). After title, abstract and full text screening and eligibility assessment, we identified 16 studies for inclusion and added three more studies from the bibliographies of these publications for a total of 19.

A data extraction form, which included 17 items (publication year, main objective(s), methodology overview, exposure data, outcome data, publication type, study design, study population (or unit), sample size, study country, geographic scope, summary of results, mental health/well-being outcomes, emotional impacts, notes about moderators/mediators/pathways, implications/recommended actions, and future research recommendations) was used to structure and support knowledge synthesis. The data charting process was conducted independently by each author and all ambiguities regarding the results and interpretation considered for the final scoping review were discussed among the authors. A short narrative summary of each of the 19 publications was written to further contribute to synthesis. Also, after completing the data charting and narrative synthesis of the 19 publications, the authors met to discuss the potential pathways between wildfire smoke exposure and mental health and well-being and to codevelop the proposed model presented in our results. The authors self-identify as a U.S. white, able-bodied man and a Canadian white settler, able-bodied woman.

## Results

The results of this scoping review represent key findings and insights relevant to the impacts of wildfire smoke on mental health and well-being. In the sub-sections below, findings from our analysis of 19 articles are presented. First, we summarize key characteristics of the publications, then we summarize and synthesize the set of studies using quantitative data followed by a summary of the qualitative, mixed methods, and literature review studies. Finally, we provide a model of the potential multiple interacting pathways between wildfire smoke exposure and mental health and well-being.

### General characteristics of the publications

Overall, we found that the literature on wildfire smoke and mental health and well-being is growing, characterized by a diversity of study designs and methods, and predominantly taking place in North America and Australia. Table 2 displays the author, date, study objective(s), study design, and mental health and well-being outcomes measured or discussed for all studies included in this review.

**Table 2:** Summary of 19 studies included in the review

Author, date	Study objective(s)	Data Collection	Study Design	Mental health and well-being outcomes measured or identified
Duclos, 1990 [39]	To evaluate selected health impacts of the 1987 Northern California forest fires.	Quantitative	Ecological (retrospective time-series)	Mental health and psychological “problems” measured as panic and/or anxiety
McDermott, 2005 [40]	To investigate wildfire-related factors and their relation to PTSD and to compare child and adolescent reports of PTSD with parent reports of postdisaster psychopathology.	Quantitative	Cross-sectional (survey)	PTSD (measured using Post Traumatic Stress Disorder Reaction Index); general psychopathology (measured using The Strengths and Difficulties Questionnaire)
Moore, 2006 [41]	To study if increases in PM2.5 and PM10 during the 2003 Kelowna/Kamloops fires were associated with changes in physician visits for specific disease categories.	Quantitative	Ecological (time-series)	Mental disorders (based on ICD diagnostic codes)
Marshall, 2007 [42]	To estimate the prevalence of major depressive disorder and PTSD among individuals who had sought disaster services or shelter as a result of a wildfire three months previously and to evaluate whether a screener might aid in identification of persons at elevated risk of subsequent psychopathology.	Quantitative	Cross-sectional (survey)	Depressive symptoms (using the eight-item version of Patient Health Questionnaire and PTSD (using the Post-traumatic Symptom Checklist)
Caamano-Isorn, 2011 [43]	To analyze the respiratory and mental health effects of the 2006 Galician wildfires, using consumption of anxiolytics-hypnotics and drugs for obstructive airway diseases as indicators.	Quantitative	Ecological (geographical- and temporal-cluster study)	General psychiatric pathology (using anxiolytics-hypnotic use as indicator)
Hazelwood Mine Fire Inquiry, 2014 [44]	To provide a chronology of key events during the Hazelwood coal mine fire, relevant to the environmental and health management of the emergency and its impacts on the community.	Mixed methods	Rapid Health Risk Assessment	Depression, anxiety, post-traumatic stress disorders, lethargy,and panic attacks. Feelings of fear, anxiety, and confusion. Increased risk of family violence, drug and alcohol abuse.
Ho, 2014 [36]	To explore the immediate physical and psychological symptoms among the general population who experienced haze during the 2013 Southeast Asian Haze Crisis and examine factors associated with the severity of acute physical and psychological symptoms.	Quantitative	Cross-sectional (online survey)	Psychological stress (measured using the Impact of Event Scale - Revised IES-R). Reports of recurrent thinking and dreams about haze, irritability, insomnia, and poor concentration.
De Pretto, 2015 [45]	To assess the links between knowledge, attitudes, and practices in relation to transboundary haze episodes related to seasonal forest fires in Southeast Asia.	Quantitative	Cross-sectional (purposive survey)	Well-being generally (adapted from Diener’s standard well-being instrument). Reports of worry, concern about health impacts of haze, sadness.
Reid, 2016 [9]	To assess the evidence of health effects from exposure to wildfire smoke (respiratory, cardiovascular, mental, and perinatal health specifically) and to identify susceptible populations.	Literature review	Systematic literature review	Psychological impacts, mental health, PTSD
Vincent, 2018 [46]	To examine the existing literature on firefighters’ sleep quantity and quality during wildland firefighting operations.	Literature review	Literature review	Depression, PTSD, stress, sleep quality and quantity
Berger, 2018 [47]	To examine the perceptions of school staff of the impact of the Hazelwood mine fire event on student and staff well-being.	Qualitative	Semi-structured interviews	Anxiety, stress, and feelings of frustration, fear and feeling overwhelmed. Reports of increased violence in homes.
Dodd, 2018 [48]	To explore the lived experience of individuals and communities affected by the 2014 wildfire season in the Northwest Territories and to examine the impact of the wildfires and smoke on mental and emotional well-being, physical health, and livelihoods.	Qualitative	Semi-structured interviews	Elevated feelings of depression, fear, hopelessness, stress and uncertainty.
Christianson, 2019 [49]	To examine residents’ wildfire evacuation experiences in Whitefsh Lake First Nation #459, Canada in May 2001 and to explore factors that complicated the evacuation process and put further strain on the evacuees and members of Whitefish Lake First Nation.	Qualitative	Semi-structured interviews	Stress, panic, and feelings of concern, confusion, worry, fear, exhaustion, recurring negative thoughts when seeing and smelling smoke.
Tan, 2019 [50]	To investigate the effect of acute short-term haze exposure on cerebral hemodynamics in healthy individuals, in addition to the relationship between haze exposure and psychosomatic symptoms.	Quantitative	Ecological	Psychological stress, psychosomatic symptoms.
Mottershead, 2020 [51]	To explore how the Dene Tha’ First Nation evacuated their community due to wildfire smoke in the summer of 2012, identify factors that helped and hindered the evacuation process, and examine how the evacuation process affected evacuees.	Qualitative	Semi-structured interviews and case study	Depression and stress (lasting/long term stress for some participants). Feelings of frustration and confusion.
Heaney, 2021 [52]	To assess the current evidence regarding optimal public communication strategies used in smoke-related disaster scenarios and best practices to connect with and empower populations to avoid exposure and health consequences associated with bushfire smoke.	Literature review	Scoping review	General psychosocial and mental health effects.
Rodney, 2021 [6]	To examine the effects of prolonged exposure to bushfire smoke-related air pollution on physical health, mental health, and sleep patterns of residents of the Australian Capital Territory region during the 2019–20 bushfire season and how these vary by demographic and lifestyle factors.	Quantitative	Cross-sectional (online survey)	Mental health symptoms, anxiety, depression, sleep.
Pelletier, 2022 [53]	To identify health research priorities for wildland firefighters and related personnel.	Mixed methods	Cross-sectional (survey) and semi-structured interviews	General mental health impacts, stress.
Humphreys, 2022 [21]	To describe how persistent smoke events impact mental health and well-being, how community members have coped with these impacts, and identify adaptation opportunities to mitigate well-being impacts in future wildfire events.	Qualitative	Focus groups and key informant interviews	Anxiety, worry, and depression. Feeling of malaise, unmotivated, helpless, and guilt.

There was an overall increasing trend in the number of publications over time, with 70% published since 2014. Although we searched for publications from 1990 onwards, we only found one published between 1990 and 2004. In terms of geography, the countries which were most represented were Australia (*n* = 4), Canada (*n* = 5), and the USA (*n* = 3) highlighting that most of the literature is situated within high-income countries. Middle-income countries represented, all located within Southeast Asia, included Singapore (*n* = 2) and Malaysia (*n* = 1); there were no low-income countries represented. Geographic scope of the publications included local/community (*n* = 6), regional (*n* = 9), national (*n* = 1) and international (*n* = 3).

Of the 19 publications, 15 were original empirical research, one was a report, and three presented findings from literature review processes. Among the original empirical research publications, nine publications collected and analyzed quantitative data, five qualitative data, and one used mixed methods [the Hazelwood Mine Fire Inquiry [44] was a report that also utilized mixed methods]. A diversity of methods and designs were used in the publications. Ecological and cross-sectional study designs using surveys were common among the quantitative publications; semi-structured interviews were used in all five of the publications that collected qualitative data.

Regarding smoke exposure data among the nine studies using quantitative methods, four publications characterized exposure based on population/community exposure to wildfire smoke events (e.g., assumed smoke exposure because of proximity to fire events), three publications used self-reported measures of exposure (e.g., self-reported wildfire exposure as none, mild or severe), and two studies used outdoor air quality monitoring data. With respect to the outcome data for the quantitative studies, most measured mental health and well-being outcomes with self-reported data (five of nine), three used administrative data (e.g., emergency room data, prescription data), and one publication used a physiological measure.

### Summary of publications using quantitative data

We reviewed nine quantitative studies. Three studies used administrative data to measure mental health outcomes [39, 41, 43]. Duclos compared emergency room visits in six California counties during a wildfire to visits during two reference periods (a year before and two weeks before the fire) [37]. There was a statistically significant increase in visits for asthma, COPD and other conditions such as laryngitis but the increase in mental health conditions was not statistically significant (*p* = 0.4). Smoke exposure was not specifically assessed or extracted from the medical record and the authors reported that air monitoring could not be quantified reliably for the exposed counties.

Moore analyzed physician billing records to study the relationship between increases in PM_2.5_ and PM_10_ from wildfires in two regions of Canada and physician visits for physical and mental health complaints [41]. There was a positive association between wildfire particulate matter and increased weekly rates of physician visits for respiratory complaints in one of the two regions. Increased rates of visits for cardiovascular and mental illnesses were detected before, during and after the fires and the authors felt they could not be ascribed to the fires. Similarly, Caamano-Isorna analyzed a database of pharmaceuticals prescribed by primary care doctors to study the association of the 2006 wildfires in Galicia, north-west Spain, with respiratory and mental health effects, using the consumption in that region of drugs for obstructive airway diseases and anxiolytics-hypnotics as indicators [43]. The study found a significant increase in anxiolytic-hypnotics consumption among men from exposed municipalities; it did not assess smoke exposure independently of fire exposure or other wildfire-related experiences.

Rodney’s study surveyed Australian residents of thirty-one postal codes that had experienced high levels of smoke-related air pollution from the 2019–2020 Australian Capital Territory bushfire season [6]. Outcomes included self-reported physical health, mental health, and sleep patterns and whether they attributed their symptoms to smoke. Participants were recruited by letters posted to homes, from a pre-existing population panel, and by social media, radio advertisements, and word of mouth. The response rate for the letter invitations was 6.4%. 45.3% reported anxiety due to the smoke and 21.4% reported feeling depressed because of the smoke. Negative mental health symptoms were more common among women and 25–54 year olds. Bivariate models disclosed greater odds of mental health symptoms among parents, persons with pre-existing physical health diagnoses, persons with pre-existing mental health diagnoses, and persons reporting direct exposure to the current fires. Over 37% of respondents reported disrupted or poor sleep which was positively associated in bivariate analyses with females, younger age groups, pre-existing physical diagnoses, direct exposure to the current fires, and direct exposure to previous bushfires.

Two studies employing a trauma mental health framework included questions about wildfire smoke exposure as an indicator of a perceived threat to the safety of participants or their families [40, 42]. McDermott conducted a study on posttraumatic stress disorder and general psychopathology in children and adolescents following wildfires in the Australian Capital Territory in 2003 [40]. Children and adolescents who saw smoke scored significantly higher on components of the Strengths and Difficulties Questionnaire, a screening instrument for child and adolescent mental health in which higher scores are predictive of psychiatric diagnoses. In the trauma mental health framework, the authors considered witnessing smoke to be an indicator of a perceived threat to the child and adolescent participants or their family, thereby affecting their stress and emotional well-being. In Marshall’s trauma mental health study of evacuees of a 2003 Southern California wildfire, difficulty breathing due to smoke or ashes along with house or property damage and physical injury to self or loved one were predictors of a probable PTSD or major depression diagnosis [42]. After adjusting for demographic characteristics, difficulty breathing due to smoke exposure was no longer a predictor of probable mental health diagnosis.

Three studies focusing on the smoke and air pollution from annual forest fires caused by agricultural land clearing techniques and burning of tropical peatland, called “haze”, reported adverse effects on mental health and well-being [36, 45, 50]. Ho studied acute psychological symptoms due to short-term exposure to haze during the 2013 Southeast Asian Haze Crisis [36]. Singapore participants were recruited during a week when the Pollution Standard Index (PSI) reached 401 on a scale of 0–500, with > 300 defined as hazardous for health. Participants were a snowball sample of mainly students aged 18–29 years old. The total on the Impact of Events Scale-Revised (IES-R) score was 18.47 (S.D. = 11.69) consistent with mild to moderate psychological stress (primarily intrusion symptoms and hyper-arousal symptoms) but below the threshold of acute stress reaction syndrome at a score of 33. The participants’ intrusion symptoms included recurrent thinking about haze, negative feelings from reminders of the haze, dreams about haze, and recurrent mental pictures of haze. The arousal scores included irritability, being easily startled, insomnia, poor concentration, and physical reactions after reminders of the haze. Perceiving a lower level of PSI as dangerous was associated with higher IES-R total scores (β = − 2.734, SE = 1.374, R^2^ = 0.013, *p* = 0.047), mean intrusion score (β = − 0.162, SE = 0.074, R^2^ = 0.016, *p* = 0.029), and mean hyperarousal score (β = − 0.217, SE = 0.087, R^2^ = 0.020, *p* = 0.013). The total number of physical symptoms was associated with the mean avoidance score (β = 0.048, SE = 0.011, R2 = 0.061, *p* < 0.001), mean intrusion score (β = 0.075,SE = 0.013, R^2^ = 0.095, *p* < 0.001), mean hyperarousal score (β = 0.075,SE = 0.013, R^2^ = 0.095, *p* < 0.001), total mean IES-R score (β = 0.080, SE = 0.011, R^2^ = 0.153, *p* < 0.001), and total IES-R score (β = 0.080, SE = 0.011, R^2^ = 0.153, *p* < 0.001).

Tan and colleagues studied the effect of haze exposure on cerebral hemodynamics also during the 2013 Southeast Asian Haze Crisis in Singapore [50]. The authors hypothesized that vasoactive substances in the haze would cause cerebral vasculature vasodilation which might lead to psychosomatic symptoms (sore throat, nausea, anxiety, insomnia, poor appetite, headache, neck stiffness, cough, sputum, breathlessness, runny nose, joint pain, rash, lethargy, itching, and watery eyes) [50]. Cerebral hemodynamics (pulsatility index, resistivity index), blood pressure, and symptoms were measured before and immediately after participants were exposed to 30 minutes of outdoor haze. Haze exposure caused modest, significant changes in cerebral hemodynamics. Moreover, participants with one or more new psychosomatic symptoms after haze exposure had pronounced changes in cerebral hemodynamics that were not observed among asymptomatic volunteers, leading the investigators to postulate a differential susceptibility to the vasoactive effects of haze on cerebral vasculature.

In the third study of haze in Southeast Asia, De Pretto et al. surveyed two sets of residents of Kuala Lumpur, Malaysia in 2014 about their knowledge, concerns, and practices in relation to haze: amateur athletes taking part in a duathlon and members of the general public in a shopping mall [45]. Concern was high in most participants: 70% of persons surveyed reported sadness in response to haze conditions, 80% reported feeling sad because of “the negative effects on the natural environment” and 79% reported being afraid for their health. Concern was significantly higher among participants from the duathlon event than among shopping mall respondents (3.94 ± 0.4 vs. 3.80 ± 0.5; *p* < 0.05). In analyses of covariance conducted to test the effect of the different variables simultaneously, parenthood (t = 2.90, df = 294, *p* < 0.05) and age (t = 3.35, df = 294, *p* < 0.05) were associated with higher concern. There was a weak positive correlation between greater knowledge about the haze and greater concerns (r = 0.15, t = 2.6, df = 300, *p* < 0.05).

### Summary of publications using qualitative data, mixed-methods and literature reviews

As outlined above, our review included five publications using qualitative data, three literature reviews, and two mixed-methods publications (one empirical study and one report). These publications provide a deeper understanding of the lived experiences of the mental health and well-being impacts of wildfire smoke exposure and a more fulsome understanding of the myriad ways that exposure to smoke may affect mental health and well-being beyond clinical diagnostic outcomes and categories.

Three of the qualitative publications examined mental health and well-being impacts in Indigenous communities in Canada and provide intriguing insights into how long periods of smoke events, along with evacuations, can affect mental health and well-being. These studies are particularly valuable given that Indigenous communities, which are commonly located in forest ecosystems prone to wildfire, are at heightened risk of exposure to wildfire and smoke events [54]. Importantly, these communities are also at risk of repeated exposures over time and cumulative impacts associated with smoke exposure along with wildfire evacuation processes and face structural and systematic racism and marginalization [49]. Mottershead et al. worked with the Dene Tha’ First Nation to explore the wildfire evacuation experience, to identify factors that helped and hindered the evacuation process, and to examine how the evacuation process affected evacuees, including mental health and well-being [51]. The study was part of a larger project and partnership called the First Nations Wildfire Evacuation Partnership and used a community-based case-study approach. Drawing on individual and group semi-structured interviews with 31 community members, authors reported that the evacuation experience and being confined indoors due to wildfire smoke led to feelings of frustration, isolation, stress, and depression for residents while they awaited updates and for the fire to be contained. Some community members noted that stress lasted beyond the evacuation and wildfire event with longer term consequences for mental health and well-being. Also noteworthy, having limited information overall and about smoke exposure and air quality beyond visual observation exacerbated mental and emotional health impacts. The authors also report that the following factors contributed to the communities’ adaptation to the wildfire event and evacuation: strong leadership, keeping families together, providing social support, and using familiar host communities.

Dodd et al’s 2018 study examined the impact of prolonged wildfire smoke events on mental health and well-being throughout the summer of 2014 in the Northwest Territories [48]. Based on 30 interviews with community members, community leadership, Elders, and other relevant key informants (e.g., local physician and public health official), the research team found that prolonged and persistent smoke exposure had wide-ranging effects on mental and emotional well-being. The majority of those interviewed reported “a direct connection between the wildfires and smoke and a decrease in their mental and emotional health.” [48]. Elevated feelings of fear, anger, depression, stress, isolation, hopelessness and uncertainty were frequently expressed. Being confined to home and disruptions to land-based activities led to isolation from community and family which exacerbated feelings of loneliness, stress, and anxiety. Personal and community isolation were the most commonly expressed consequences associated with living through the “summer of smoke” [48]. Persons who depended on the land for supplementing their food also experienced increased food insecurity. Relatedly, lack of outdoor physical activity was a mental health stressor and the physical symptoms of smoke exposure impeded such activities. The emotional and mental impacts of being separated from the land, outdoor activities, food sources and livelihoods, along with dislocation due to evacuations, are succinctly described by research participants in the following quotations and align with the concept of solastalgia:

*It was the lost summer…the attachment to the land and place, what it does, and when you get alienated, you know, from that place…it takes a deep, emotional toll, if not a spiritual toll*

As another respondent said:

*It was like we didn’t have a summer, for me, because, usually we get outside, we do things on the water…we enjoy being in the North. We enjoy being outside. We enjoy the environment. We enjoy cooking. Everything that’s outside, we enjoy, and, I feel like I lost that…that impacts you emotionally and mentally*

Findings also illustrate community resilience [conceptualized as how communities avoid, reduce, or cope with the damages caused by disasters, and how they recover with minimal social disruption] and a strong sense of community in response to the prolonged wildfire events, smoke exposure, and wildfire evacuations.

Christianson et al. also conducted semi-structured interviews (*n* = 35) to learn about experiences of evacuations due to a wildfire and smoke event that occurred in May 2011 in Whitefish Lake First Nation 459 (Alberta, Canada) [49]. Although most mental health and well-being consequences reported in this publication were linked specifically to the community evacuation process, participants shared that exposure to smoke heightened feelings of concern, fear, and distress. Seeing smoke in the sky, which is very common in summer months for Whitefish Lake residents, triggered emotional impacts and feelings of concern and fear after returning to the community underscoring the recurring and lasting effects [49]. Several studies in our review describe how persons experiencing wildfire events had recurring negative thoughts about their experiences when confronted with reminders like the smell of smoke and images of smoke [40, 47, 49, 55].

Humphreys et al. used focus groups and interviews to describe how extreme and persistent smoke events impact mental health and well-being in rural communities in Washington state (USA) [21]. Participants discussed a diversity of emotional impacts and responses associated with persistent smoke events including worry, stress, guilt, depression, lack of motivation, hopelessness, and helplessness. Interestingly, those who left the community during smoke events also reported feeling stress as well as guilt. The authors argue that the impacts of wildfire smoke on mental health and well-being may be particularly problematic in rural communities, which tend to be “dependent on contributions of nature to people’s quality of life” [21]. When discussing pathways leading to anxiety, stress, and depression, key factors included experiencing physical health effects (such as respiratory effects), isolation, and lack of physical exercise. This study also examined how community members coped with these impacts and possible actions to mitigate well-being impacts in future wildfire events. Findings highlighted the need for stress reduction activities and support groups, a community clean air space to facilitate gathering and address social isolation, resources dedicated to strengthening social connections in rural communities, community air quality monitoring, and free air filters (particularly for low-income groups).

Heaney, Vincent, and Reid reported on literature review processes related to wildfire smoke and mental health and being [9, 46, 52]. Heaney synthesized current evidence pertaining to optimal public communication strategies used in smoke-related disasters using a scoping review methodology [52]. The authors reviewed 67 articles that focused on health communication and/or adverse health effects of smoke exposure in the context of wildfire (bushfire) smoke events. Only 8 of 67 articles in the review made the connection between wildfire smoke events and psychosocial and mental health effects specifically. The authors emphasize that smoke exposure is usually associated with mild psychological distress, that isolation is a common precipitating factor, and that effective health communication may help reduce mental health consequences [52]. They also highlight that “... it is difficult to differentiate the psychological impacts specifically related to smoke exposure from other potentially traumatizing factors like forced evacuation from home, an approaching fire front, or the loss of loved ones and belongings”.

The literature review from Reid and colleagues [9], aimed to assess the evidence of health effects from exposure to wildfire smoke (respiratory, cardiovascular, mental, and perinatal health specifically) and to identify susceptible populations using a modified systematic review methodology (based on Woodruff and Sutton) [56]. Studies that were reviewed were assessed for risk of bias based on considerations of sample size, study exposure assessment methods, controlling for potential confounding factors, and use of objective outcome measures. They found six studies that investigated the association between wildfire smoke exposure and objective mental health impacts and outcomes. Two studies were assessed as low potential for bias (which we also describe above); Duclos [39] and Moore [41]. The review highlighted the lack of rigorous research on the association between wildfire smoke and mental health outcomes and highlighted the “lack of information about which populations are most susceptible to wildfire smoke exposure and associated health impacts”.

Vincent et al’s literature review focused on wildland firefighter sleep and associated impacts on safety and health (including mental health) illustrating that wildland firefighters who are constantly exposed to smoke, noise and heat may experience impaired sleep quality and quantity which may have physiological and cognitive effects [46]. Pelletier also focused specifically on the health of wildland firefighters [53]. This study used a survey and semi-structured interviews with wildland firefighters and related personnel to identify health research priorities among this subgroup with heightened vulnerability to health impacts of wildfire smoke. The effects of exposures on fatigue and sleep, stress, and overall mental health (78%) were identified as key research priorities, along with long term health impacts generally.

Finally, two publications included in our review focused on the Hazelwood Mine Fire*,* a 45 day-long mine fire sparked by a nearby bushfire which led to persistently elevated air pollution and dramatic, thick plumes of ash and smoke covering nearby towns [44]*.* The Hazelwood Mine Fire Inquiry used a Rapid Health Risk Assessment approach and gathered qualitative and quantitative data to generate a comprehensive after event report and to examine the health effects that the smoke and ash produced [44]. Due to the fire and smoke event, families had to move away, wear masks and staying indoors to reduce smoker exposure. Also, schools relocated and businesses closed down. The assessment of psychological impacts stated:

*Many community members have developed levels of anxiety and depression, which they attribute to the mine fire. Issues raised by community members at community consultations included concern about evident smoke and ash and the generally unpleasant environment during the mine fire, and also the unknown long-term impact of the mine fire to their health. A number of individuals advised that they were afraid to leave their home for the period of time that the mine fire was burning. Many residents also suffered anxiety and stress from disrupted family life, the loss of enjoyment of their home and neighborhood, the smell in the air, and because they could not go outside…. The Board also heard evidence about the broader social effects of the Hazelwood mine fire. Concerns were expressed during community consultations about the potential for an increase in family violence in the short to medium-term as a result of stress caused by the mine fire. Professor Campbell advised the Board that the whole community, especially young children, are at risk of psychosocial impacts as a result of the emergency, including an increased risk of family violence, drug and alcohol abuse, depression and anxiety, post-traumatic stress disorders and phobias.* (Part 4, page 318)

The Hazelwood Mine Fire Inquiry emphasized that the community was highly concerned about the long-term physical and mental health impacts of smoke and ash exposure associated with the fire [44]. A phenomenological analysis of school relocation due to the Hazelwood Mine Fire by Berger et al. supports the report’s findings and describes elevated anxiety among students and increases in stress and violence in the home [47].

### Pathways between wildfire smoke exposure and mental health and well-being

**Fig. 2:**
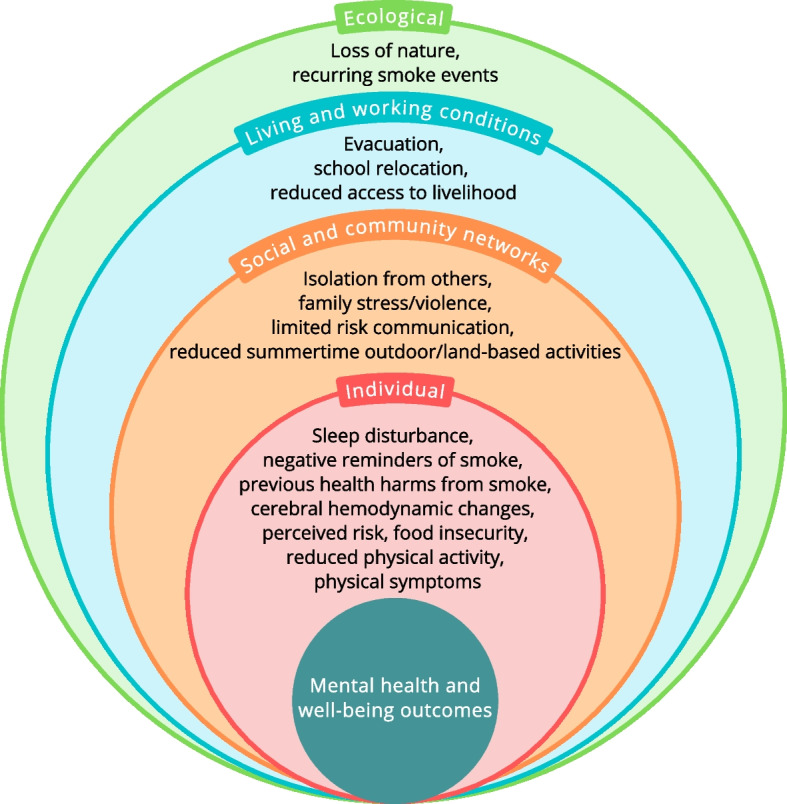
Potential pathways between wildfire smoke exposure and mental health and well-being

The publications we reviewed provide insights into underlying pathways which may connect smoke exposure with adverse mental health and well-being. We identified potential mechanisms operating at several levels including the Individual, Social and community networks, Living and working conditions, and Ecological levels (Fig. 2) These levels are nested and interactively influence mental health and well-being. Mechanisms can cross between multiple levels and can influence mental health and well-being differently, based on cumulative and intersectional experiences. The interplay and interaction between these mechanisms may be complex and vary in different communities, people, and situations. Our model is adapted from Lawrance and colleagues [57], which was based on Dahlgren and Whitehead, Bronfenbrenner’s Ecological Systems Theory and the Lancet Commission for Global Mental Health and Sustainable Development [58, 59, 60]. The model is based on limited evidence since the number of publications in our review is small and a heterogeneous mix of populations, outcomes, and study types. However, it illustrates the need for future research to consider the variety of mechanisms and interconnected levels by which wildfire smoke may harm mental health and well-being.

Individual level mechanisms include physiological and physical mechanisms such as diminished sleep quantity and quality [6, 46, 53], changes in cerebral perfusion [50], physical symptoms from the smoke [6, 21, 36, 48], food insecurity [48], and reductions in outdoor physical activity [48]. The individual level also includes psychological and emotional mechanisms, such as previous negative physical harms from the smoke, perceived risk of danger [36], and negative reminders from smoke [36, 40, 47, 49, 55]. These are nested in social and community network factors including isolation from people [21, 46, 48, 51, 52] and reduced summertime outdoor activity brought on by smoke [48], the potential for family stress and violence that may occur [44], and risk communications from authorities that may be limited or inadequate [36, 51, 52].

The individual level and social and community level factors are placed in the context of living and working conditions such as home evacuations [6, 26, 49, 49, 51, 55], school relocations [44, 47], and reductions in access to one’s livelihood [21, 44, 48] particularly for outdoor workers and people in the recreation and tourism industries. All of this occurs in the ecological context of increasing and possibly yearly, repeated and prolonged smoke events and the feeling of a loss of nature that occurs [21, 44, 48]. As authors have noted, it is challenging to tease out the mental health impact of exposure to wildfire smoke from the impact of evacuation and traumatic experiences such as an approaching fire front and lack of information or poor communication from government agencies [6, 52]. Though these proposed pathways arose from our review they must be considered preliminary and hypothetical.

## Discussion

We provide a scoping review examining how wildfire smoke may affect mental health and well-being. With coverage of the global, quantitative and qualitative literature spanning outcomes, our study offers the most comprehensive review of research on the mental health and well-being consequences and pathways to date.

Compared to the understanding of the physical health impacts of wildfire smoke exposure, the understanding of the mental health impacts of wildfire smoke is in its infancy. Our review suggests that exposure to wildfire smoke may have mental health impacts, particularly in episodes of chronic and persistent smoke events, but the evidence is inconsistent and limited. The knowledge and evidence gaps alongside the growing risks of exposure and impacts underscore the importance of additional research in this realm.

Reviewing the quantitative research, overall, we concur with Reid that methodological limitations make it difficult to reach a conclusion from these studies [9]. Studies of comparable events such as seasonal haze do find an association [36, 45, 50] but also have a likelihood of bias due to their non-random samples. Studies applying a trauma framework do not provide sufficient information to assess the association [40, 42]. Moreover, it is difficult to tease out the differential impacts of exposure to wildfire smoke from other frightening and traumatic experiences such as witnessing fire or an evacuation. Short term exposures like those studied by Moore and Duclos may not have a mental health impact [39, 41]. Also, using administrative health data may limit detection in rural communities where population density and both mental healthcare seeking and access are low.

The quantitative empirical studies were further limited by their methods of exposure determinations and measurement of mental health outcomes. It is challenging to accurately assess exposure, and many studies relied on self-reported exposure or duration of time exposure. For example, a limitation of both Moore’s and Caamano-Isorno’s studies is that the exposure classifications are crude and non-specific [41, 43]. All residents in the population are assumed to be equally exposed during the fire period which is not likely the case. None of the quantitative studies adequately examined the characteristics differentiating vulnerable and susceptible sub-groups though Reid’s literature review study did aim to identify susceptible populations [9]. Also relevant, the long-term mental health and well-being impacts of wildfire smoke, and the impacts of exposure to multiple events, are inadequately understood in the existing literature.

Qualitative studies provide a richer sense of the topic and a more fulsome understanding of lived experiences. In Dodd’s study conducted after a wildfire season with over 40 days of smoke, affected residents far from the fires said it was like they lost their summer [48]. People reported isolation from others, less community participation, loss of nature, and worsened mental health.

The learning from qualitative research allows a more nuanced understanding of the social well-being, emotional well-being and spiritual impacts of wildfire smoke events and exposures. Qualitative research provides outcomes of importance beyond diagnostic codes and categories which are inherently limited in information and meaning, and likely less prevalent. It provides an opportunity for cultural framing of mental health. By avoiding the pathologizing framework of mental health diagnoses it also allows more stakeholders to engage, for instance first responders and community members who may not want to be pathologized as traumatized [42]. Qualitative research is well suited to community participatory research methods and greater community ownership over the research process and results. Understanding wildfires, particularly smoke but any of the issues examined here, as lived experiences invites greater discourse.

Our model of the potential pathways between smoke exposure and mental health and well-being effects is a contribution that scientists can use to connect the lessons of past studies with future research. The mechanisms proposed are supported by research in similar fields uncovering the biological and non-biological channels through which non-wildfire related air pollution leads to mental health problems. The population mental health effects of urban fine particulate air pollution (PM_2.5_ and PM_10_) from industrial and motor vehicle emissions is documented [61, 62]. A study in Belgium found that the association between air pollution derived PM_2.5_ and generalized anxiety disorder was partially mediated by a difference in physical activity and Wang reported the association between PM_2.5_ and depression was mediated by physical activity [30, 36]. Inhaled pollution activates biological pathways implicated in the development of depression [61, 62] and wildfires may have worse toxicities; for example, the particulate matter in wildfire smoke may be more toxic to lung tissue than particulate matter from ambient air pollution [63, 64]. While concentrations of fine particulate matter from U.S. anthropogenic sources are decreasing due to reduced industrial emissions, climate model simulations predict a 55% increase in wildfire-related PM_2.5_,—making the study of wildfire smoke’s mental health effects all the more important [8]. As regulations seek to further reduce anthropogenic sources of emissions, wildfire smoke may become the major source of ambient air pollution in regions of the continental United States [8, 65]. Air pollution studies also suggest that psychological factors can moderate vulnerability to the negative effects of environmental pollutants on health and physiology [64, 66, 67]. We suggest that living under the lockdowns of the COVID-19 pandemic may have given readers some sense of what this is like; the isolation from community and the dread that leaving the house to go into the world outside is fundamentally dangerous might sum up the isolating and fearsome experience of the pandemic and persistent wildfire smoke events.

### Implications for future research

Our review suggests many priorities for future research. We recommend more rigorous methods to generate more robust conclusions about the mental health and well-being impacts of wildfire smoke exposure. For example, improved epidemiological studies that accurately measure individual and population smoke exposure are needed to better characterize specific health impacts. The reviewed wildfire research used self-reported exposure or spatially and temporally resolved models of exposure to outdoor wildfire smoke levels as proxies for exposure, without accounting for the effects of pollutant transfer indoors. Advances in the measurement of wildfire smoke and air quality are needed to address the existing limitations in exposure assessment [68]. Wildfire smoke exposure accounting for indoor and outdoor chemical and particulate matter concentrations can improve this exposure characterization. Emerging technologies in ground-level monitoring of air quality alongside innovations in wearable sensors that reduce the potential of misclassification bias in exposure assessment present opportunities for addressing existing gaps and limitations in the existing research. Another priority is research differentiating between mental illness or probable mental illness on the one hand and emotional well-being on the other. Quality of life should also be studied as an outcome. Current research is mostly from the global north (United States, Canada, Australia) with only three studies published from the global south. Studies conducted in the global south are urgently needed where fuel types may differ, susceptible populations may differ, fires frequently extend for long times, and interventions to protect mental health and well-being may differ [69]. For example, seasonal “haze” is created by wildland fires due to slash-and-burn techniques for clearing agricultural lands in South Asia, where the cultural framing of mental health and well-being may differ compared to the global north and where mental health resources are in short supply.

At the same time, more work is needed to identify the contextual factors that set the stage for mental health and well-being effects and the pathways and mechanisms that link wildfire smoke to mental health and well-being effects. Our proposed pathways model can be used to inform future data collection investigating how smoke harms mental health and well-being. Since reducing the factors and their interactions may also positively influence mental health, the model can be used to understand which protective factors mitigate against mental health and well-being harms. Still, missing from the research is anything more than a preliminary understanding of which pathways may be important in different populations and contexts. This includes identifying susceptible populations and a better understanding of the mechanisms and risk factors underlying the associations. Additional contextual factors that should be considered are socioeconomic disadvantage, marginalization, and housing attributes that influence the indoor accumulation of wildfire smoke. The specific groups studied in the papers we reviewed included fire-fighters, Indigenous communities (in Canada), and residents of rural communities. Further research is needed to uncover other mechanisms and mediators that may explain mental health and well-being effects in other groups, such as age (particularly children) and gender groups. Finally, studies to date have focused mainly on the Individual level and Social and community network level. Additional factors that may influence risk and impact such as experiences of systemic racism and community cohesion should be considered in future research.

The accelerating increase in wildfires and population smoke exposures calls for new priorities and approaches, too. Wildfires are becoming chronic, multi-week events, as was seen in Australia in 2019 and California in 2020 [21]. Residents of rural areas in Washington state (USA) call the summer wildfire smoke season the “Fifth Season” “when smoke from wildfires blankets North Central Washington for weeks or even months at a time.” [70] As outlined in the Hazelwood Mine Fire inquiry, the long term adverse effects of exposure to smoke, including mental health impacts, are of great concern to the community [44]. This parallels studies on emotional distress brought on by chronic industrial and vehicular air-pollution. But the only studies that considered chronic exposure have been qualitative; longitudinal studies particularly of cumulative and chronic exposures are required [44, 47, 48, 51].

Finally, we found only one study that addressed community response to chronic wildfire smoke [21]. Needed are studies identifying actions, strategies, and interventions individuals, families and communities use to cope with wildfire smoke events such as community gathering spaces, community monitoring, and clean air spaces [21]. Participatory research, that engages with at-risk and affected communities throughout the research process in meaningful ways, is particularly well-suited to identifying solutions and interventions. Understanding the mediators of mental health effects can be useful to intervention development too. For example, Ho suggests in their study of the psychological impact of haze that reducing physical symptoms from smoke and addressing its perceived danger through updates, education and regular communications may help reduce psychological stress [36]. Motterhead similarly found that limited official communications about smoke exposure and air quality exacerbated mental and emotional health impacts, further underscoring the need for updated communications (and community based air quality monitoring) [51]. More rapid research conducted during and directly after events can address these topics.

Although this paper presents results from a comprehensive, transparent, and repeatable scoping review process, there are limitations that should be recognized when interpreting the findings and considering implications. First, our review only searched for and therefore only includes English language papers. This may have influenced our finding papers mainly from the countries of the global north. Similarly, only a few studies centered the perspectives of Indigenous First Nation communities so the results reflect primarily a Western-centric, high-income country perspective of mental health and well-being. We set the start date for our search in 1990 due to constraints of time and resources for the review. Including earlier years in our literature search could add to the available data and enrich the results. However, our inclusion of only one paper from the decade 1990 to 2004 suggests that papers before 1990 are unlikely. Any variability in the outcomes we found may be influenced by the heterogeneity of the populations studied, variability of the wildfire smoke exposures, and the range of health outcomes studied. Also, because we used a scoping review process, we did not assess the quality of articles included in our review [37]. The qualitative research we reviewed provided a contextual exploration of the possible pathways, still we recognize that these study findings may not be generalizable to all communities.

## Conclusion

We provide the first scoping review ever conducted examining how wildfire smoke may affect mental health and well-being. We identified potential pathways for mental health and well-being impacts that exist on multiple levels. These pathways that we organized in a nested model can guide researchers in their future data gathering. They provide a conceptual framework for future knowledge synthesis efforts in this topic. Also, they may be helpful to public health, community mental health, and emergency management practitioners seeking to mitigate the harms of wildfire smoke on mental health and well-being. Future studies should focus on the chronic, persistent or repeated smoke events. More research is required beyond those of richer Northern countries and across cultures with different understandings of mental health, available resources, and needs for intervention. Well-being and quality of life should also be studied as an outcome. This can be addressed best in large longitudinal cohort studies that monitor the trajectories and long-term impacts of susceptible populations. The data and key findings of this report highlight gaps in the literature and suggest areas for theoretical, methodological, and empirical advances for wildfire smoke and mental health research.

## Data Availability

The datasets used and analyzed during the current study are available from the corresponding author on reasonable request.

## References

[1] Flannigan MD, Stocks BJ, Wotton BM (2000). Climate change and forest fires. Sci Total Environ.

[2] Westerling AL. Wildfire Simulations for California’s Fourth Climate Change Assessment: Projecting Changes in Extreme Wildfire Events with a Warming Climate. California’s Fourth Climate Change Assessment, California Energy Commission. 2018. Publication Number: CCCA4-CEC-2018-014. https://www.energy.ca.gov/sites/default/files/2019-11/Projections_CCCA4-CEC-2018-014_ADA.pdf. Accessed 27 Oct 2022.

[3] Jolly WM, Cochrane MA, Freeborn PH, Holden ZA, Brown TJ, Williamson GJ (2015). Climate-induced variations in global wildfire danger from 1979 to 2013. Nat Common.

[4] Abatzoglou JT, Williams AP (2016). Impact of anthropogenic climate change on wildfire across western US forests. Proc Natl Acad Sci U S A.

[5] Jones MW, Smith A, Betts R, Canadell JG, Colin Prentice I, QuéréC L (2020). Climate change increases the risk of wildfires.

[6] Rodney RM, Swaminathan A, Calear AL, Christensen BK, Lal A, Lane J (2021). Physical and mental health effects of bushfire and smoke in the Australian Capital Territory 2019-20. Front Public Health.

[7] Sapkota A, Symons JM, Kleissl J, Wang L, Parlange MB, Ondov J (2005). Impact of the 2002 Canadian forest fires on particulate matter air quality in Baltimore city. Environ Sci Technol.

[8] Ford B, Val Martin M, Zelasky SE, Fischer EV, Anenberg SC, Heald CL (2018). Future fire impacts on smoke concentrations, visibility, and health in the contiguous United States. GeoHealth.

[9] Reid CE, Brauer M, Johnston FH, Jerrett M, Balmes JR, Elliott CT (2016). Critical review of health impacts of wildfire smoke exposure. Environ Health Perspect.

[10] Le G, Breysse P, McDermott A, Eftim S, Geyh A, Berman J (2014). Canadian forest fires and the effects of long-range transboundary air pollution on hospitalizations among the elderly. ISPRS Int J Geoinf.

[11] O’Dell K, Bilsback K, Ford B, Martenies SE, Magzamen S, Fischer EV (2021). Estimated mortality and morbidity attributable to smoke plumes in the United States: not just a western US problem. GeoHealth..

[12] Liu JC, Wilson A, Mickley LJ, Ebisu K, Sulprizio MP, Wang Y (2017). Who among the elderly is most vulnerable to exposure to and health risks of fine particulate matter from wildfire smoke?. Am J Epidemiol.

[13] Borchers Arriagada N, Horsley JA, Palmer AJ, Morgan GG, Tham R, Johnston FH (2019). Association between fire smoke fine particulate matter and asthma-related outcomes: systematic review and meta-analysis. Environ Res.

[14] Linares C, Carmona R, Tobías A, Mirón IJ, Díaz J (2015). Influence of advections of particulate matter from biomass combustion on specific-cause mortality in Madrid in the period 2004-2009. Environ Sci Pollut Res Int.

[15] Sahani M, Zainon NA, Wan Mahiyuddin WR, Latif MT, Hod R, Khan MF (1994). A case-crossover analysis of forest fire haze events and mortality in Malaysia. Atmos Environ.

[16] Reid CE, Jerrett M, Tager IB, Petersen ML, Mann JK, Balmes JR (2016). Differential respiratory health effects from the 2008 northern California wildfires: a spatiotemporal approach. Environ Res.

[17] Kondo MC, De Roos AJ, White LS, Heilman WE, Mockrin MH, Gross-Davis CA (2019). Meta-analysis of heterogeneity in the effects of wildfire smoke exposure on respiratory health in North America. Int J Environ Res Public Health.

[18] Cascio WE (2018). Wildland fire smoke and human health. Sci Total Environ.

[19] Davies IP, Haugo RD, Robertson JC, Levin PS (2018). The unequal vulnerability of communities of color to wildfire. Plos one.

[20] Shrestha PM, Humphrey JL, Carlton EJ, Adgate JL, Barton KE, Root ED, Miller SL (2019). Impact of outdoor air pollution on indoor air quality in low-income homes during wildfire seasons. Int J Environ Res Public Health.

[21] Humphreys A, Walker EG, Bratman GN, Errett NA (2022). What can we do when the smoke rolls in? An exploratory qualitative analysis of the impacts of rural wildfire smoke on mental health and wellbeing, and opportunities for adaptation. BMC Public Health.

[22] Hanigan IC, Johnston FH, Morgan GG (2008). Vegetation fire smoke, indigenous status and cardio-respiratory hospital admissions in Darwin, Australia, 1996-2005: a time-series study. Environ Health.

[23] Rosenthal N, Benmarhnia T, Ahmadov R, James E, Marlier ME (2022). Population co-exposure to extreme heat and wildfire smoke pollution in California during 2020. Environ Res: Climate.

[24] Disler R, Glenister K, Wright J (2020). Rural chronic disease research patterns in the United Kingdom, United States, Canada, Australia and New Zealand: a systematic integrative review. BMC Public Health.

[25] McGee TK, Nation MO, Christianson AC (2019). Residents’ wildfire evacuation actions in Mishkeegogamang Ojibway Nation, Ontario, Canada. Int J Disaster Risk Reduct.

[26] Lelieveld J, Evans JS, Fnais M, Giannadaki D, Pozzer A (2015). The contribution of outdoor air pollution sources to premature mortality on a global scale. Nature..

[27] Dictionary of Psychology. American Psychological Association, Washington. 2020. https://dictionary.apa.org/mental-health. Accessed 7 July 2022.

[28] To P, Eboreime E, Agyapong VIO (2021). The impact of wildfires on mental health: a scoping review. Behav Sci (Basel).

[29] Pun VC, Manjourides J, Suh H (2017). Association of ambient air pollution with depressive and anxiety symptoms in older adults: results from the nshap study. Environ Health Perspect.

[30] Wang R, Liu Y, Xue D, Yao Y, Liu P, Helbich M (2019). Cross-sectional associations between long-term exposure to particulate matter and depression in China: the mediating effects of sunlight, physical activity, and neighborly reciprocity. J Affect Disord.

[31] Power MC, Kioumourtzoglou MA, Hart JE, Okereke OI, Laden F, Weisskopf MG (2015). The relation between past exposure to fine particulate air pollution and prevalent anxiety: observational cohort study. BMJ..

[32] Min JY, Kim HJ, Min KB (2018). Long-term exposure to air pollution and the risk of suicide death: a population-based cohort study. Sci Total Environ.

[33] Casas L, Cox B, Bauwelinck M, Nemery B, Deboosere P, Nawrot TS (2017). Does air pollution trigger suicide? A case-crossover analysis of suicide deaths over the life span. Eur J Epidemiol.

[34] Ng CFS, Stickley A, Konishi S, Watanabe C (2016). Ambient air pollution and suicide in Tokyo, 2001–2011. J Affect Disord.

[35] Hautekiet P, Saenen ND, Demarest S, Keune H, Pelgrims I, Van der Heyden J (2022). Air pollution in association with mental and self-rated health and the mediating effect of physical activity. Environ Health.

[36] Ho RC, Zhang MW, Ho CS, Pan F, Lu Y, Sharma VK. Impact of 2013 south Asian haze crisis: study of physical and psychological symptoms and perceived dangerousness of pollution level. BMC Psychiatry 2014;14(1):81. http://dx.doi.org/10.1186/1471-244X-14-81 10.1186/1471-244X-14-81 PMC3995317 24642046

[37] Arksey H, O’Malley L (2005). Scoping studies: towards a methodological framework. Int J Soc Res Methodol.

[38] Levac D, Colquhoun H, O’Brien KK (2010). Scoping studies: advancing the methodology. Implement Sci [Internet].

[39] Duclos P, Sanderson LM, Lipsett M. The 1987 forest fire disaster in California: assessment of emergency room visits. Arch Environ Health. 1990;45(1):53–8. 10.1080/00039896.1990.9935925 2180383

[40] McDermott BM, Lee EM, Judd M, Gibbon P (2005). Posttraumatic stress disorder and general psychopathology in children and adolescents following a wildfire disaster. Can J Psychiatr.

[41] Moore D, Copes R, Fisk R, Joy R, Chan K, Brauer M (2006). Population health effects of air quality changes due to forest fires in British Columbia in 2003: estimates from physician-visit billing data. Can J Public Health.

[42] Marshall GN, Schell TL, Elliott MN, Rayburn NR, Jaycox LH (2007). Psychiatric disorders among adults seeking emergency disaster assistance after a wildland-urban interface fire. Psychiatr Serv.

[43] Caamano-Isorna F, Figueiras A, Sastre I, Montes-Martínez A, Taracido M, Piñeiro-Lamas M (2011). Respiratory and mental health effects of wildfires: an ecological study in Galician municipalities (north-West Spain). Environ Health.

[44] Hazelwood Mine Fire Inquiry. 2014. https://www.parliament.vic.gov.au/file_uploads/8101_HAZ_Hazelwood_Mine_Inquiry_Report_BOOK_LR_f5Bp6wNh.pdf. Accessed 11 Feb 2021.

[45] De Pretto L, Acreman S, Ashfold MJ, Mohankumar SK, Campos-Arceiz A (2015). The link between knowledge, attitudes and practices in relation to atmospheric haze pollution in peninsular Malaysia. Plos one.

[46] Vincent GE, Aisbett B, Wolkow A, Jay SM, Ridgers ND, Ferguson SA (2018). Sleep in wildland firefighters: what do we know and why does it matter?. Int J Wildland Fire.

[47] Berger E, Carroll M, Maybery D, Harrison D (2018). Disaster impacts on students and staff from a specialist, trauma-informed Australian school. J Child Adolesc Trauma.

[48] Dodd W, Scott P, Howard C, Scott C, Rose C, Cunsolo A (2018). Lived experience of a record wildfire season in the Northwest Territories, Canada. Can J Public Health.

[49] Christianson AC, TK MG, Whitefish Lake First Nation 459 (2019). Wildfire evacuation experiences of band members of Whitefish Lake First Nation 459, Alberta, Canada. Nat Hazards (Dordr).

[50] Tan BY, Leong AZ, Leow AS, Ngiam NJ, Ng BS, Sharma M (2019). Psychosomatic symptoms during south east Asian haze crisis are related to changes in cerebral hemodynamics. Plos one.

[51] Mottershead KD, McGee TK, Christianson A (2020). Evacuating a first Nation due to wildfire smoke: the case of Dene Tha’ first Nation. Int J Disaster Risk Sci.

[52] Heaney E, Hunter L, Clulow A, Bowles D, Vardoulakis S (2021). Efficacy of communication techniques and health outcomes of bushfire smoke exposure: a scoping review. Int J Environ Res Public Health.

[53] Pelletier C, Ross C, Bailey K (2022). Health research priorities for wildland firefighters: a modified Delphi study with stakeholder interviews. BMJ Open.

[54] Erni S, Johnston L, Boulanger Y, Manka F, Bernier P, Eddy B, et al. Exposure of the Canadian wildland–human interface and population to wildland fire, under current and future climate conditions. Can J For Res. 51(9):1357–67. 10.1139/cjfr-2020-0422.

[55] Camilleri P, Healy C, Macdonald E, Nicholls S, Sykes J, Winkworth G, et al. Recovery from bushfires: the experience of the 2003 Canberra bushfires three years after. Australas j paramed. 2010;8(1). 10.33151/ajp.8.1.112.

[56] Woodruff TJ, Sutton P (2014). The navigation guide systematic review methodology: a rigorous and transparent method for translating environmental health science into better health outcomes. Environ Health Perspect.

[57] Lawrance EL, Thompson R, Newberry Le Vay J, Page L, Jennings N (2022). The impact of climate change on mental health and emotional wellbeing: a narrative review of current evidence, and its implications. Int Rev Psychiatry.

[58] Dahlgren G, Whitehead M. Policies and strategies to promote social equity in health. Background document to WHO - Strategy paper for Europe. Institute for Futures Studies. 2007. https://core.ac.uk/download/pdf/6472456.pdf. Accessed 27 Oct 2022.

[59] Bronfenbrenner U. Ecological systems theory. In: Making human beings human: bioecological perspectives on human development. Thousand Oaks: Sage Publications Ltd; 1992. p. 106–73.

[60] Patel V, Saxena S, Lund C, Thornicroft G, Baingana F, Bolton P (2018). The lancet commission on global mental health and sustainable development. Lancet.

[61] Van den Bosch M, Meyer-Lindenberg A (2019). Environmental exposures and depression: biological mechanisms and epidemiological evidence. Annu Rev Public Health.

[62] Wegesser TC, Pinkerton KE, Last JA (2009). California wildfires of 2008: coarse and fine particulate matter toxicity. Environ Health Perspect.

[63] Franzi LM, Bratt JM, Williams KM, Last JA (2011). Why is particulate matter produced by wildfires toxic to lung macrophages?. Toxicol Appl Pharmacol [Internet].

[64] Splete H. Pollution levels linked to physical and mental health problems. Medscape. 2022. ; https://www.medscape.com/viewarticle/970482?uac=198810HX&faf=1&sso=true&impID=4118277&src=mkm_ret_220327_mscpmrk_psych_mental.

[65] Kaulfus AS, Nair U, Jaffe D, Christopher SA, Goodrick S (2017). Biomass burning smoke climatology of the United States: implications for particulate matter air quality. Environ Sci Technol.

[66] Miller JG, Gillette JS, Manczak EM, Kircanski K, Gotlib IH (2019). Fine particle air pollution and physiological reactivity to social stress in adolescence: the moderating role of anxiety and depression. Psychosom Med.

[67] Dzhambov AM, Markevych I, Tilov B, Arabadzhiev Z, Stoyanov D, Gatseva P (2018). Pathways linking residential noise and air pollution to mental ill-health in young adults. Environ Res.

[68] Kelleher S, Quinn C, Miller-Lionberg D, Volckens J (2018). A low-cost particulate matter (PM2.5) monitor for wildland fire smoke. Atmospheric. Meas Tech.

[69] Johnston FH, Henderson SB, Chen Y, Randerson JT, Marlier M, Defries RS (2012). Estimated global mortality attributable to smoke from landscape fires. Environ Health Perspect.

[70] Watts LH (2022). Telling the story of wildfire smoke risks.

